# Supplementary material for: The Specific Heat of Astro-materials: Review of Theoretical Concepts, Materials, and Techniques
Source: Int J Thermophys. 2022 Aug 1;43(9):144. doi: 10.1007/s10765-022-03046-5 (PMC9343321; doi:10.1007/s10765-022-03046-5)
Supplement: Supplementary file 1 — Supplementary file1 (DOCX 802 kb) [file 10765_2022_3046_MOESM1_ESM.docx]

**Appendix (Supplementary Information)**

**The specific heat of astro-materials:
Review of theoretical concepts, materials and techniques**

Jens Biele^(1)^, Matthias Grott^(2^), Michael E. Zolensky^(3)^, Artur Benisek^(4)^, Edgar Dachs^(4)^

*^(1)^ DLR – German Aerospace Center, RB-MUSC, 51147 Cologne, Germany, e-mail: Jens.Biele@dlr.de*

*^(2)^ DLR – German Aerospace Center, Institute for Planetary Research, Berlin, Germany*

*^(3)^ NASA Johnson Space Center (Houston, United States)*

*^(4)^* *Chemistry and Physics of Materials, University of Salzburg, 5020 Salzburg, Austria.*

1. **Methods**

## Accurate Padé approximants to the Debye and Kieffer functions

For the **Debye function[[1]](#footnote-1)**, we approximate, following Goetsch et al. [1],

$$D(\theta_{D}/T)=3\left(\frac{T}{\theta_{D}}\right)^{3}\int_{0}^{\theta_{D}/T} \frac{x^{4}e^{x}}{(e^{x}-1)^{2}}dx=3\left(\frac{T}{\theta_{D}}\right)^{3}\int_{0}^{\theta_{D}/T} \frac{x^{4}e^{-x}}{(e^{-x}-1)^{2}}dx$$

with the rational function (Padé approximant) *g*(*T_n_*), *T_n_*=*T*/θ*_D_*

$$g(T_{n})=\frac{N_{0}+\frac{N_{1}}{T_{n}}+\frac{N_{2}}{T_{n}^{2}}+\cdots}{D_{0}+\frac{D_{1}}{T_{n}}+\frac{D_{2}}{T_{n}^{2}}+\cdots}$$

The first one, two or three and the last one, two or three in each of the sets of coefficients *N_i_* and *D_i_* in *g*(*T_n_*) can be chosen to exactly reproduce both the low- and high-*T* limiting values and power-law dependencies in *T* and/or 1/*T* of the function it is approximating; this is a very important and powerful feature of the Padé approximant. Then the remaining terms in powers of 1/*T_n_* in the numerator and denominator have freely adjustable coefficients that are chosen to fit the intermediate temperature range of the function. A physically valid approximant requires that there are no poles of the approximant on the positive real *T_n_* axis.

The constraints which assure the low-*T* and high-*T* limiting values and derivatives here are [1],

$$\begin{matrix} D_{0}=\frac{N_{0}}{E}, D_{1}=\frac{N_{1}}{E}, D_{1}=\frac{-FN_{0}+EN_{2}}{E}, D_{m}=\frac{N_{n}}{G} \\ E=3, F=-3/20, G=12\pi^{4}/5 \\ m=n+3 \end{matrix}$$

Result: The approximant in [1] has n=5 and m=8 and a maximum relative deviation of <0.3%; increasing the degrees to n=8, m=11, we obtain a maximum relative deviation to true Debye =
5.8⋅10^-6^ at *T*/θ*_D_* ≈ 0.1 (Table 2 and Figure 1)

Table 1 Coefficients for Padé-approximant of the Debye function

N_0_..N_8_:

3

-0.17682974

0.019953909

0.00065686146

4.0944374e-05

1.3642663e-06

4.4605133e-07

-1.2957876e-08

1.6782041e-09

D_0_..D_11_:

**1**

**-0.058943245**

**0.056651303**

-0.0027325104

0.0010646772

-3.2134724e-05

7.3396614e-06

7.0397353e-08

7.2090357e-09

1.8991184e-09

-5.540749e-11

**7.1785057e-12**

The coefficients in bold numbers are not independent. Thus, 17 independent coefficients remain. The denominator-polynomial D has no zeros at positive temperatures. The Padé fit has been compared to the exact Debye function, calculated by precise numerical integration and with the function in the polylogarithmic form (the polylogarithm function, with double and optionally arbitrary precision is available in Matlab^(TM)^).

Debye function, polylogarithm form

x=θ_D_/T, *Li_s_(x)* is the polylogarithm of order *s* and argument *x*.

$$\begin{matrix} C_{V}/3R=\frac{4}{5}\frac{\pi^{4}}{x^{3}}+\frac{3x\exp(-x)}{\exp(-x)-1}+12\ln\left[1-\exp(-x)\right] \\ -\frac{36}{x}Li_{2}\left[\exp(-x)\right]-\frac{72}{x^{2}}Li_{3}\left[\exp(-x)\right]-\frac{72}{x^{3}}Li_{4}\left[\exp(-x)\right] \end{matrix}$$

**Figure 1 Relative deviations of our Padé-fit from the true Debye-function**

**Padé-approximant for the Kieffer-function**

Gurevich et al. [2], equation 5 for the *C_P_* of crystal hydrate water needs the Kieffer-function

$$K(\theta_{L}/T,\theta_{U}/T)\equiv\frac{3R}{\theta_{L}/T-\theta_{U}/T}\int_{\theta_{L}/T}^{\theta_{U}/T} \frac{x^{2}\exp(x)}{\left(\exp(x)-1\right)^{2}}dx$$

which has two arguments, lower and upper normalized temperatures.

The following Padé-approximant with numerical coefficients from Table 2 is accurate to <1⋅10^-6^ in relative terms over arbitrary arguments (Figure 2):

$$\begin{matrix} k(x)=\frac{1+\sum_{n=1}^{8} p_{n}x^{n}}{1+\sum_{m=1}^{9} q_{m}x^{m}} \\ K(x_{L},x_{U})=\frac{3R}{x_{U}-x_{L}}\left[x_{U}k(x_{U})-x_{L}k(x_{L})\right] \\ x_{L}=\theta_{L}/T, x_{U}=\theta_{U}/T \end{matrix}$$

Table 2 Coefficients for Padé-approximant of Kieffer-function. Note that both *q_9_* = 1 (exactly) and *p_8_*= π²/3 (exactly). Furthermore, *p*_(1...3)_ = *q*_(1...3)_.

| ***n, m*** | ***p_n_*** | ***q_m_*** |
| --- | --- | --- |
| 1 | **-291.060215932407**  **1278381.54701115**  **114269.722348969** | |
| 2 |
| 3 |
| 4 | 22596.3244579461 | 58062.5772985723 |
| 5 | 2635.11630655455 | 5887.48838373051 |
| 6 | 337.259940890294 | 821.72512559065 |
| 7 | 7.47974537995418 | 102.316628131888 |
| 8 | **π²/3** | 2.27417852421094 |
| 9 |  | **1** |


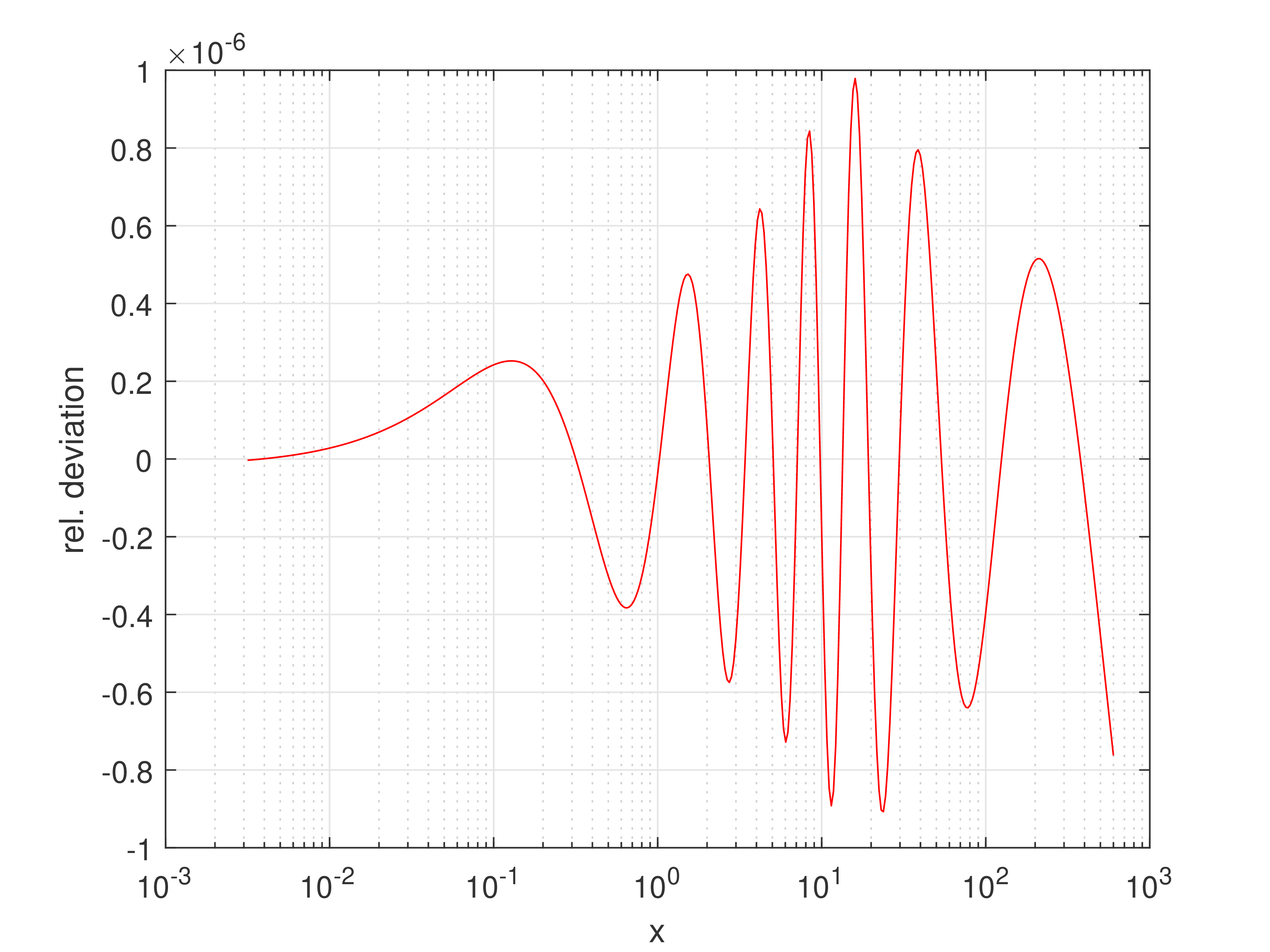


Figure 2 Relative deviations of our Padé-fit from the true Kieffer-function k(x)

## Estimating the mineral composition from *c_p_*(*T*)

The basic idea is the following (C, c here designate both specific heat capacities at zero pressure in J/kg/K):
Given the experimental *c_P_*-curve (*T*; *c_P_*) of a mineral mixture over a (wide as possible) temperature range and some idea about the main constituents (endmember minerals), i.e. a list of endmember minerals. Main means: mass fraction of a constituent >>1%. We estimate the ‘most likely’ mass fractions *X_i_* of the assumed or known constituent minerals by least squares solution of the constrained cp mixing equation (cp_decompose) and then, with the obtained mass fractions that are best (i.e., with the least χ²) reproducing the weighted cp data, construct the model *c_P_*(*T*) curve over a wider temperature range (cp_compose) and calculate the uncertainties of the fitted an extrapolated values both by Monte Carlo and a bootstrap [3] method. Note that the endmember mineral *c_P_*(*T*) curves are the base functions in our least-squares problem here; they are generally far from been orthogonal, and the problem is only solvable because of the constraint that $X_{i}\geq0, \sum_{i} X_{i}=1$.
Given *M* experimental data points, $T_{m},C_{m}(T_{m}), m=1\cdots M$ , we fit the mass fractions *X_i_* using as base functions the *c_i_(T)* of *N* possible constituent minerals, since $C_{m}(T_{m})=\sum_{i} X_{i}c_{i}(T_{m})$ subject to the constraints $X_{i}\geq0, \sum_{i} X_{i}=1$ . Let *σ* be the uncertainties of the *C* data (weighting). This is a linear least-squares problem with bounds and linear constraints, in typical solver lingo (e.g,, Matlab’s solver lsqlin) it is written:
$\min_{X} \frac{1}{2}\left\|C\cdot X-d\right\|_{2}^{2} \text{such that }\left\{\begin{matrix} A_{eq}X=b_{eq} \\ lb\leq X\leq ub \end{matrix}\right.$

with

$$\begin{matrix} C=\left[\begin{matrix} c_{1}(T_{1})/\sigma_{1} & c_{2}(T_{1})/\sigma_{1} & \ldots& c_{N}(T_{1})/\sigma_{1} \\ c_{1}(T_{2})/\sigma_{2} & c_{2}(T_{2})/\sigma_{2} & \ldots& c_{N}(T_{2})/\sigma_{2} \\ \ldots& \ldots& \ldots& \ldots\\ c_{1}(T_{M})/\sigma_{M} & c_{2}(T_{M})/\sigma_{M} & \ldots& c_{N}(T_{M})/\sigma_{M} \end{matrix}\right] \\ \\ X=[X_{1},X_{2},\ldots,X_{N}]^{T} \\ d=[C_{1}/\sigma_{1},C_{2}/\sigma_{2},\ldots,\mathrm{C}_{M}/\sigma_{M}]^{T} \\ A_{eq}=\mathbf{1} (1\times N \text{vector}) \\ \mathrm{b}_{eq}=1 \\ lb=\mathbf{0} (1\times N \text{vector}) \\ ub=\mathbf{1} (1\times N \text{vector}) \end{matrix}$$

Because the problem being solved is always convex, the solver finds a global, although not necessarily unique, solution. It is highly recommended, if non-trivial constraints on the mineral composition are known, they should be furnished as bounds (*lb, ub*) for *X*.

**Uncertainty estimate of the solution:** By default we do both a Monte Carlo (varying the *d* vector with an assumed random noise + bias within error bounds of e.g., 1%) and a bootstrap resampling method to calculate the uncertainty distribution of the resulting *X_i_* . Sufficiently small *X_i_* can also be set identical to 0 and the process repeated. An alternative to Monte Carlo with an assumed random noise + bias is bootstrap resampling. Monte Carlo and bootstrap give rather similar results.

A number of *X_i_* can be grouped for convenience (belonging to the same mineral group or solution series, like fraction of fayalite and forsterite in olivine, or or-al-an in feldspars, or diopside-hedenbergite-enstatite-ferrosilite in pyroxene. We still have to solve the equation system above, but can then disentangle the *X_i_* into groups:

$$\begin{matrix} C_{m}=\sum_{j=1:J} W_{j}\sum_{k=1:K(j)} w_{k}c_{j,k}(T_{m}) \\ \sum W_{j}=1, W_{j}\geq0 \\ \sum w_{k}=1, w_{j}\geq0 \\ X=[W_{1}w_{1},W_{1}w_{2},\ldots W_{2}w_{1},\ldots W_{J}w_{K}] \\ \sum X_{k}=1 \end{matrix}$$

Convention: call *J* the number of *W*; *K* is a vector containing the number of *w* in each *W_j_*.

**Example: lunar regolith data**

First we checked that a synthetic *T*, *c_P_, σ*(*c_P_*) set (with known *X*) is correctly fitted and the result is identical to the known, true *X* (to within ~1⋅10^-5^ relative or 6⋅10^-7^ in absolute mass fractions). The code can check after the first, nominal, fit whether any components of the result *X* are <*X*_threshold_ (typically 0.001); it deletes the corresponding mineral(s) from the list, and re-performs the fit with the reduced minerals list. Then, we perform a Monte Carlo (~1000 runs) where each time we either do a bootstrap or add noise to the *d=c_P_/σ(c_P_)*-vector: nominally, our model noise is one third (0.58% if total uncertainty is the default 1%) of gaussian noise, one third of a linear bias with Gaussian random amplitude, one third of quadratic bias with Gaussian random amplitude. The result can be inspected in histograms (binned vs. mass fraction for each retained mineral), Figure 27.

**Figure 3 Monte Carlo histogram of fitted mineral compositions for synthetic lunar regolith. Red dotted: nominal fit without noise. Magenta: mean; Black: median; blue: mode; bold green: true value. Calculated with lb=0 and ub=1 for each X, Xthreshold=0.001; MC=50000**

# Data review for meteorite sample specific heat data

Only a handful of heat capacities of meteorites have been published until about ten years ago, when interest in astro-material thermophysical properties, including heat capacity, suddenly increased. The new meteorite *c_P_* data are, to our present knowledge, [4-13] and more are in preparation (e.g., Noyes, Macke, Opeil et al., "Novel Low Temperature Behavior in the Thermal Diffusivity and Thermal Inertia of Iron Meteorites" (submitted, 2022) including *c_P_*(*T*) over the range 2-300 K for four different iron meteorites [R. Macke, Jan 15, 2022, pers. comm.]).

The more interesting recent works start with Opeil et al. (2012) [4] who determined, besides thermal conductivity of various meteorite samples, also *c_P_(T)* for Martian shergottite meteorite Los Angeles Los Angeles: [14, 15] from ∼2 to 380 K by PPMS.

Macke et al. (2014) [6, 7] measured heat capacity at 175 K for several HEDs (diogenites, eucrites, howardites). Macke et al. (2016) [8] provided PPMS data on heat capacities of ordinary chondrite falls (i.e., less likely weathered than finds) in the range (75–300) K, later extended to 5 - 300 K [11]. Opeil et al. (2020) determined thermal properties, including *c_P_* by PPMS, of five CM2 meteorites in the range 5 - 300 K [12].

Very interesting is the work of Piqueux et al. (2021) [13] who measured the specific heat of ~30 different meteorites of many classes from ~90 to 285 K by DSC, fairly accurately (~5% accuracy).

Virtually all of the older data were measured at temperatures at or above ~300 K, we describe them briefly hereunder.

Butler (1963) [16] reported the specific heat curve of octahedrite meteoritic iron from Canyon Diablo meteorite fall for 123 to 570 K (uncertainty unknown).

Matsui and Osako (1979) [17] directly measured the heat capacity of five Yamato meteorites (four ordinary chondrites and a howardite) at 300 K, 350 K, and 400 K, while Yomogida and Matsui (1983) [18] used laboratory data of the constituent minerals of ordinary chondrites to calculate their heat capacities; their calculated values, which they preferred, were 50% higher than their directly measured results. According to Yomogida & Matsui, this is very likely not due to any significant discrepancies between theoretical (linear mixing) and measured values but a bias introduced by sample preparation in the data of [17] (grinding leading to a bias in metal concentration).

Soini, Kukkonen et al. (2020) [19] compile the older *c_P_* data, including some modelled heat capacities, of a large number of meteorites plus new simultaneous contactless thermal conductivity and thermal diffusivity measurements, from which *c_P_* can be calculated to an accuracy of at best 5% (yet higher scatter from same samples measured in orthogonal directions with rather different *c_P_*, up to 25% difference!). Even for class averages (at only 2 temperatures, 200 K and 300 K), the scatter is high (there are finds and fall meteorites in the list, i.e. potential weathering, as discussed above, and various petrographic grades), but the trend is *c_P_*(LL)~*c_P_*(C)>*c_P_*(L)>*c_P_*(H)>*c_P_*(IAB), with the mesosiderite somewhere in between.

Beech et al. 2009 [20] measured the *c_P_* of a H5 chondrite Gao-Guenie fragment. Specific heat values have been determined over the temperature range between 296 and 773 K; furthermore, for Gao-Guenie (07C-TPRL) – H5, Gao-Guenie (08) – H5, Jilin-H5 and Sikhote-Alin-Iron IIAB the specific heat at ~350 K has been determined by ‘A standard water immersion calorimetry technique’, resulting in 732.0 ± 7.5, 739.7 ±27.5, 725.8 ±13.2 and 458.2 ±10.7 J/kg/K, respectively. Data might be problematic according to Flynn, Consolmagno et al. 2018 [21].

Szurgot et. al (2011) [22] determined, with DSC and a ‘double-walled calorimeter’ the specific heat at ~297 K of various samples of Brahin, Vaca Muerta, Allende, El Hammami, Gold Basin, Sahara 99471, DaG 610, Canyon Diablo, Gibeon, Sikhote Alin, Toluca and Morasko meteorites.

Opeil and Consomagno [23] noted that the heat capacity of chondritic meteorites is typically ~750 J/kg/K at 300 K, ~500 J/kg/K at 200 K.

(Łuszczek and Wach 2014) [24] determined *c_P_* of crust and interior of L-type ordinary chondrite NWA 6255 (petrographic type L5, shock stage S4, weathering grade W1) by DSC, 223-823 K. It contains a few % (1.0% in crust, 3.6% in interior) of troilite FeS, its transition peak is seen in the *c_P_* curves.

Flynn, Consolmagno et al. 2018 [21] report in their review on ‘Physical properties of the stone meteorites’ some problematic measurements, citing, for instance, Matsui and Osako (1979), Yomogida and Matsui, (1983) which we discussed above, but also Beech et al. (2009) [20].

We briefly add to this list, hereunder, a few instances where we found issues:

The specific heat of the Morasko iron meteorite, from Szurgot et al. (2008) [25], from 263 to 303 K, must be grossly in error. They report, in a figure and in text, that the specific heat capacity *c_P_* varies from 50 to 590 J/kg/K in this temperature range, which we believe is impossible. The literature value is a variation from 428 to 449 J/kg/K over this small temperature range.

Occasionally, the temperature of a specific heat value is not quoted correctly (or not at all), probably stemming from the assumption that temperature is not important; for example, (Yu and Ji 2015) [26] estimate *c_P_* of Bennu (B-type, analogue likely CM meteorites as ~500 J/kg/K or 560 J/kg/K, no temperature given, referring to Opeil 2012 [27] . These values appear very small! In the Opeil (2012) paper, we find ~750 J/kg/K at 300 K; the 560 J/kg/K value comes actually from Gundlach and Blum (2013) [28] ; 500 J/kg/K is a realistic CM value for 175 K, the average temperature[[2]](#footnote-2) in Consolmagno & Macke’s LN2 experiments.

Ghosh and McSween (1999) [29] studied, theoretically, the temperature dependence of specific heat capacity and its effect on asteroid thermal models. Their *c_P_*(*T*) curves, 300-1100 K (their fig. 1) are grossly in error. They note that ‘there are no measurements on ferrosilite, albite, orthoclase, or diopside’ - they must have overlooked it, precise data on said minerals exist at least since the 1980ies.

Henke (2012) [30] calculate cp of H and L ordinary chondrites based on an assumed composition taken from the literature ([18, 31] and heat capacities from Barin (1995) [32]. However, the calculated curves might be in error and the said references are outdated.

Szurgot and Wach (2012) [33] give the *c_P_* of the ordinary chondrite L6 Sołtmany meteorite with ~5% troilite (an equilibrated ordinary chondrite L6 class) and the Gao-Guenie chondrite; the data look problematic, at least for Sołtmany.

# Data review for the 9 lunar samples where *c_P_* was measured

Note that the sample number prefix for Apollo samples is 10 for Apollo-11, 12 for Apollo-12, 14 for Apollo-14, 15 for Apollo-15, 6 for Apollo-16 and 7 for Apollo-17. Major geologic features and rock types sampled are listed in Table 3.

Table 3 Context: Apollo missions, major geologic features and rock types (source: NASA)

| **Apollo** | **Major Geologic Features and Rock Types** |
| --- | --- |
| 11 | Mare (Sea of Tranquillity), basaltic lava |
| 12 | Mare (Ocean of Storms), rocks are basaltic lava; ray from Copernicus Crater crosses the site. |
| 14 | Highlands (Fra Mauro formation) - thought to be ejecta from the Imbrium Basin |
| 15 | Mare (Hadley Rille in a mare area near the margin of Mare Imbrium) and highlands (Apennine Mountains, a ring of the Imbrium basin); rocks are breccia and basalt |
| 16 | Highlands (Descartes formation and Cayley Plains); rocks are anorthosite and highlands soil. |
| 17 | Mare (Sea of Serenity) and Highlands; rocks are mare soil, orange soil, basaltic lava, anorthosite. |

Detailed descriptions of all lunar regolith samples have been published online by the Lunar and Planetary Institute (LPI), see <https://www.lpi.usra.edu/lunar/samples/#petrographic>.

**Table 4** gives an overview about the lunar samples for which cP (or thermal diffusivity) has been measured.

**Table 4 Overview lunar cp samples**

| **Sample** | **Description** | **Reference, notes** |
| --- | --- | --- |
| 14321,153 | Breccia, Big Bertha, Apollo 14. | [34] |
| 15555,159 | Soil, Apollo 15 | [34] |
| 14163,186 | Soil, Apollo 14 | [34] |
| 15301,20 | Soil, Apollo 15 | [34] |
| 60601,31 | Soil, Apollo 16 | [34] |
| 10057 | Type A vesicular basalt, Apollo 11 | [35] |
| 10084 | Regolith fines, Apollo 11, | [35] |
| 10021,41 | Breccia, Apollo 11 | [36] |
| 12018,84 | Olivine-dolerite, Apollo 12 | [36] |
| 10020 | Only composition: Pyroxene 45.4%, plagioclase 24.6%, olivine 3.9%, ilmenite 22.7%, troilite 0.9%, other 0.7%, void 1.8% (volume fractions) | [37]  (thermal diﬀusivity of four Apollo 11 lunar specimens measured over the temperature range -130°C to + 150°C) |
| 10046 | Only composition: Pyroxene 16.8%, Opaquest (~ilmenite) 8.6%, plagioclase 4.7%, unidentified 3.5%, glass 2.9%, matrix <40 µm (approximated by 30% glass, 55% pyroxene, 15% plagioclase) 63.5% (volume fractions) | [37]  (thermal diﬀusivity of four Apollo 11 lunar specimens measured over the temperature range -130°C to + 150°C) |
| 10017 and | (type A) | [40] LHe temperatures |
| 10046 | (type C) | [40] LHe temperatures |

The average mineral composition of lunar regolith material can be seen in **Table 5** [38]:

**Table 5 Adopted average mineral composition of lunar surface materials**

| *ρ_mix_*=3.14 g/cm³ | **Mineralogy**  **(if solid solition, mole-% fractions)** | **Vol-%** | **Density**  **g/cm³** | **Mass-%** |
| --- | --- | --- | --- | --- |
| Plagioclase | 10:90 albite/anorthite | 32 | 2.736 | 27.9 |
| Pyroxene | 40Mg60Fe mix of enstatite and ferrosilite, but ~25% Ca. i.e. a half towards 40diopside-60hedenbergite | 39 | 3.4 | 42.3 |
| Glass | Plagioclase glass with Fe:  51% SiO_2_, 24% Al_2_O_3_, 11% FeO, 14% CaO  Or 2 types:   - Mare glass, mostly basalt (pyroxenes) - Highland glass, mostly anorthosite | 19  8.5 11 | 2.9 | 17.5  8 10 |
| Olivine | ~45:55 forsterite (Mg-endmember: Mg_2_SiO_4_) and fayalite (Fe-endmember: Fe_2_SiO4) | 7 | 3.5 | 7.8 |
| Ilmenite | FeTiO_3_ | 3 | 4.74 | 4.5 |
| Troilite | FeS |  |  | 1 |
| Native iron | elemental iron-nickel metal with typically 5.7% Ni |  |  | 0.3±0.15 |

Cremers, 1974 [39] reported the Apollo *c_P_* data and also further data from lunar samples. Unfortunately, because of lacking temperature information, a single measurement on a third breccia sample (10065) by Bastin *et al.* and measurements of the Soviet Luna-16 sample reported by Vinogradov and Avduevskii *el al.*. are not really useful for us.

Morrison and Norton (1970) [40] measured the specific heat of Apollo 11 sample 10017 (type A) and 10046 (type C) at liquid helium temperatures (Table 9). The specific heat of sample 10017 increases monotonically from 19 to 28 J/kg/K in the temperature range between 2.34 K and 4.97 K. The specific heat of sample 10046, measured on the temperature range between 3.08 and 4.05 K, ranges from 10 J/kg/K to 19 J/kg/K with a maximum at 3.54 K. These values of specific heats are two orders of magnitude larger than those expected from elastic properties of these samples (Debye crystals) - they can be explained by the high fraction of amorphous constituents (glass) in the samples. The mineral composition of 10017 is rather well known, see Table 6 below.

Table 6 approximate mineral composition of lunar sample 10017 [derived from Apollo 11 Lunar Sample Information Catalog Publication: JSC-12522, https://curator.jsc.nasa.gov/lunar/catalogs/apollo11/10017.pdf]

| **Mineral** | **volume fraction, normalized to 100%, in %** | **mass fraction (calculated), in %** |
| --- | --- | --- |
| En | 8.9 | 8.1 |
| Fs | 2.2 | 2.5 |
| Di | 35.5 | 33.2 |
| Hd | 8.9 | 9.3 |
| An | 18.4 | 14.5 |
| Ab | 4.7 | 3.5 |
| Or | 0.5 | 0.4 |
| Ilm | 20.4 | 27.9 |
| Tro | 0.4 | 0.6 |

and the resulting *c_P_* is well described, up to 5.5 K, by *c_P_*(cryst) = 0.00266×*T³* [J/kg/K]. The amorphous excess, $\Delta c_{P}=c_{P}-c_{P}(cryst.)$ is shown in Figure 4, it is indistinguishable from the measured *c_P_.*

Figure 4 Excess *c_P_* of lunar samples 10017 (blue circles) and 10046 (red stars) after Morrison & Norton (1970). 10046 is a typical breccia rock with a relatively high glass content; 10017 is a vesicular basalt 10017. The results for the rock 10017 are more extensive and more accurate because the equilibrium time after heating was much shorter for
that rock (<0.5 min compared with 10 to 15 min for rock 10046), consistent with their bulk structures (10046 being more porous and cracked, its thermal conductivity 10 to 100 times smaller than that of rock 10017)

All Apollo *c_P_* data for the temperature range ~90 K to ~340 K are listed in **Table 7** and graphically shown in **Figure 5**.

If we subtract a rather arbitrary fit through each of the data sets individually, we get the scatter plot of relative differences (fit-measured)/measured in **Figure 6**, indicating experimental uncertainties of the order of max ±2%.

**Figure 5 Data overview: all published Apollo specific heat data. Marker indicates data set number used in this paper.**

**Figure 6 scatter of lunar *c_P_* data wrt. a smooth fit (equation indicated in figure) for each set. Legend indicates data set number used in this paper. Standard deviation is ~0.5%.**

The data on specific heat of Apollo samples are scattered in several publications, some are smoothed, some are not. To facilitate convenient access, we reproduce the published 9 datasets (unsmoothed) hereunder.

**Table 7 Overview: Apollo samples specific heat datasets. n is the dataset number used in this paper. 1 cal = 4.1840 J (thermochemical calorie)**

| **n** | **Reference** | **Notes** |
| --- | --- | --- |
| 1 | Robie&Hemingway, 1970 [35] | unsmoothed; *T*(K), cp (cal/g/K)  10057 (vesicular basalt) |
| 2 | Robie&Hemingway, 1970 [35] | unsmoothed; *T*(K), cp (cal/g/K)  10084 (regolith) |
| 3 | Robie&Hemingway, 1971 [36] | unsmoothed; *T*(K), cp (cal/g/K)  10021,41 (breccia) from Tranquillity Base. |
| 4 | Robie&Hemingway, 1971 [36] | unsmoothed; *T*(K), cp (cal/g/K)  12018,84 olivine dolerite from the Sea of Storms |
| 5 | Hemingway&Robie, 1973 [34] | 14163,186 (>1 mm fines) from Fra Mauro, cp in cal/g/K |
| 6 | Hemingway&Robie, 1973 [34] | 14321,153 (breccia) from Fra Mauro, cp in cal/g/K |
| 7 | Hemingway&Robie, 1973 [34] | 15301,20 (soil) from Hadley-Apennine Base, cp in cal/g/K |
| 8 | Hemingway&Robie, 1973 [34] | 15555,159 (basalt) from Hadley-Apennine Base, cp in cal/g/K |
| 9 | Hemingway&Robie, 1973 [34] | 60601,31 (soil) from Lunar Highland, cp in J/g/K |

[34-36] also reported specific heat data (Figure 30) that were smoothed using a form of least-squares orthogonal polynomials. The accuracy of the data is quoted as ±0.4 percent which of course does not include *c_P_* differences from one sample to the other due to compositional variations. The latter are seen more clearly in **Figure 7**.

**Figure 7** Lunar *c_P_*. (a) Data from 9 smoothed datasets (symbols) and mean (line) with 1σ standard deviation bars.
(b) relative deviations of 9 smoothed datasets (symbols) from the polynomial fit of Hemingway&Robie [34], with 0.4% stated uncertainty of each data point errorbar. Fit (J/kg/K) = polyval([9.6552e-8 -7.3699e-5 1.5009e-2 2.127 -23.173],*T*)
Note that datasets 1-3, 5-6 values at 90 and 360 K are extrapolated thus uncertain; dataset 4 values at 90, 340 and 360 K are extrapolated thus uncertain..

# Data tables

## Apollo lunar samples specific heat

**Table 8 Numerical data, specific heat (J/kg/K). Reference for dataset number: see Table 7. cal converted into J.**

| **T (K)** | **cP** | **T (K)** | **cp** | **T (K)** | **cp** | **T (K)** | **cp** |
| --- | --- | --- | --- | --- | --- | --- | --- |
| **dataset #1** | | **dataset #3** | | 196.75 | 577.8 | 303.96 | 777.8 |
| 103.54 | 274.4704 | 98.52 | 258.5712 | 205.69 | 591.6 | 310.91 | 784.7 |
| 111.50 | 295.8088 | 106.64 | 287.0224 | 211.38 | 605.8 | 306.01 | 778.2 |
| 120.38 | 324.6784 | 115.66 | 318.4024 | 219.94 | 625.1 | 315.65 | 795.8 |
| 129.30 | 352.2928 | 124.71 | 348.9456 | 228.32 | 640.6 | 325.09 | 812.5 |
| 137.57 | 379.4888 | 134.06 | 378.2336 | 236.92 | 658.6 | 334.25 | 828.0 |
| 145.76 | 405.4296 | 144.60 | 411.2872 | 245.66 | 674.0 | 343.42 | 845.2 |
| 154.41 | 432.2072 | 149.77 | 423.4208 | 254.37 | 689.1 | 352.89 | 861.9 |
| 163.10 | 457.7296 | 155.34 | 439.7384 | 259.68 | 699.6 | 363.10 | 876.5 |
| 171.35 | 483.6704 | 161.04 | 462.3320 | 268.92 | 717.1 | **dataset #8** | |
| 179.25 | 506.2640 | 166.52 | 472.7920 | 278.72 | 734.7 | 83.56 | 208.8 |
| 186.82 | 530.1128 | 170.23 | 496.6408 | 288.37 | 753.1 | 94.05 | 241.4 |
| 194.29 | 548.9408 | 180.38 | 517.9792 | 297.86 | 767.3 | 103.71 | 271.5 |
| 201.87 | 568.1872 | 190.50 | 540.5728 | 304.91 | 778.2 | 113.58 | 303.8 |
| 209.68 | 583.6680 | 200.17 | 565.2584 | 314.89 | 792.0 | 124.82 | 338.9 |
| 217.74 | 601.2408 | 215.92 | 600.4040 | 324.70 | 805.0 | 136.51 | 374.9 |
| 225.75 | 616.7216 | 224.86 | 616.3032 | 334.30 | 818.4 | 143.07 | 394.6 |
| 231.55 | 632.6208 | 233.44 | 631.3656 | 346.40 | 828.4 | 154.35 | 427.6 |
| 239.78 | 645.1728 | 242.6 | 650.6120 | **dataset #6** | | 165.24 | 458.6 |
| 247.68 | 659.8168 | 253.10 | 669.4400 | 96.45 | 242.7 | 176.18 | 487.9 |
| 255.59 | 675.7160 | 264.05 | 691.1968 | 104.15 | 268.2 | 187.06 | 518.0 |
| 263.63 | 688.6864 | 274.64 | 707.9328 | 112.15 | 293.7 | 197.83 | 545.6 |
| 271.93 | 703.3304 | 284.9 | 725.5056 | 120.49 | 322.6 | 208.68 | 573.6 |
| 280.23 | 717.5560 | 294.84 | 743.4968 | 129.14 | 355.2 | 218.95 | 598.3 |
| 288.28 | 730.1080 | 302.83 | 753.5384 | 133.46 | 376.1 | 230.05 | 623.0 |
| 293.21 | 738.0576 | 311.81 | 766.0904 | 143.74 | 408.4 | 240.98 | 648.1 |
| 301.01 | 748.0992 | 320.58 | 779.0608 | 154.73 | 438.9 | 249.20 | 664.8 |
| 309.16 | 762.3248 | 329.14 | 792.4496 | 165.55 | 474.0 | 260.22 | 687.4 |
| 317.53 | 770.6928 | 337.33 | 805.8384 | 168.91 | 487.4 | 271.21 | 708.4 |
| 325.72 | 782.4080 | **dataset #4** | | 177.58 | 506.7 | 281.80 | 733.0 |
| 333.76 | 791.6128 | 96.05 | 233.0488 | 185.96 | 525.5 | 293.09 | 755.6 |
| 341.59 | 804.5832 | 104.40 | 262.7552 | 194.47 | 545.6 | 303.77 | 770.3 |
| 348.44 | 813.7880 | 112.88 | 293.2984 | 203.13 | 564.8 | 313.96 | 789.5 |
| **dataset #2** | | 121.69 | 323.4232 | 211.49 | 584.5 | 323.97 | 807.1 |
| 95.17 | 268.6128 | 127.19 | 345.5984 | 220.06 | 602.5 | 333.68 | 822.2 |
| 99.56 | 277.8176 | 134.76 | 369.4472 | 228.81 | 620.5 | 343.36 | 839.7 |
| 105.16 | 291.6248 | 141.92 | 392.0408 | 237.36 | 638.5 | 353.28 | 856.5 |
| 112.25 | 312.9632 | 148.73 | 412.9608 | 245.74 | 654.4 | 363.53 | 872.8 |
| 119.98 | 335.9752 | 149.06 | 413.7976 | 252.74 | 667.3 | **dataset #9** | |
| 127.19 | 358.1504 | 158.61 | 441.8304 | 260.94 | 682.8 | 83.57 | 221.8 |
| 134.50 | 381.9992 | 167.66 | 466.9344 | 269.55 | 697.1 | 94.97 | 257.7 |
| 142.83 | 401.6640 | 176.30 | 490.3648 | 278.32 | 712.1 | 105.29 | 290.4 |
| 152.61 | 445.1776 | 184.58 | 512.1216 | 287.10 | 727.6 | 115.24 | 323.0 |
| 162.69 | 473.2104 | 193.14 | 534.7152 | 295.90 | 741.4 | 125.39 | 356.9 |
| 167.02 | 478.6496 | 201.98 | 556.0536 | 304.55 | 754.8 | 135.83 | 388.3 |
| 176.27 | 507.1008 | 210.50 | 577.8104 | 308.97 | 764.0 | 146.65 | 420.1 |
| 185.10 | 528.0208 | 218.75 | 597.8936 | 317.81 | 779.5 | 157.86 | 451.9 |
| 193.60 | 548.5224 | 222.65 | 607.9352 | 327.13 | 795.8 | 168.95 | 483.3 |
| 202.94 | 574.4632 | 231.20 | 627.1816 | 336.96 | 811.7 | 179.61 | 512.1 |
| 213.07 | 594.1280 | 240.23 | 648.5200 | **dataset #7** | | 189.82 | 535.6 |
| 222.84 | 615.0480 | 249.38 | 666.5112 | 83.98 | 221.8 | 201.17 | 564.4 |
| 224.85 | 614.6296 | 258.64 | 686.1760 | 95.46 | 256.9 | 213.39 | 595.4 |
| 229.00 | 619.2320 | 267.60 | 704.1672 | 104.47 | 287.0 | 224.23 | 620.9 |
| 231.49 | 627.1816 | 276.60 | 720.0664 | 114.25 | 319.7 | 234.21 | 643.9 |
| 233.93 | 627.6000 | 285.60 | 739.3128 | 125.20 | 349.8 | 244.40 | 665.3 |
| 239.78 | 641.8256 | 293.03 | 746.4256 | 135.82 | 382.4 | 254.90 | 687.8 |
| 246.27 | 651.0304 | 301.98 | 765.6720 | 146.20 | 417.1 | 265.60 | 710.0 |
| 253.83 | 664.4192 | 310.70 | 781.9896 | 156.58 | 444.3 | 274.91 | 728.9 |
| 262.18 | 680.7368 | 319.59 | 796.6336 | 167.16 | 472.4 | 285.53 | 751.9 |
| 270.31 | 695.3808 | 328.64 | 808.7672 | 177.64 | 500.4 | 296.10 | 771.5 |
| 278.22 | 710.4432 | **dataset #5** | | 188.03 | 528.0 | 304.06 | 789.5 |
| 285.93 | 721.3216 | 93.34 | 251.0 | 198.34 | 554.0 | 313.63 | 806.3 |
| 293.46 | 726.7608 | 101.77 | 282.0 | 208.28 | 577.4 | 323.47 | 823.0 |
| 300.84 | 739.7312 | 104.64 | 291.2 | 217.89 | 600.4 | 333.23 | 840.6 |
| 308.07 | 749.7728 | 112.07 | 318.0 | 227.13 | 620.5 | 343.17 | 857.7 |
| 327.71 | 783.6632 | 125.80 | 370.7 | 237.00 | 642.7 | 353.09 | 876.1 |
| 332.70 | 794.5416 | 133.79 | 395.0 | 247.16 | 663.6 | 363.04 | 891.6 |
| 337.93 | 797.8888 | 142.28 | 422.2 | 257.76 | 686.2 |  |  |
| 343.42 | 802.4912 | 152.82 | 448.1 | 268.32 | 706.3 |  |  |
|  |  | 169.10 | 501.0 | 278.68 | 727.6 |  |  |
|  |  | 177.70 | 521.7 | 289.07 | 748.1 |  |  |
|  |  | 187.22 | 548.5 | 299.50 | 766.9 |  |  |

Table 9 LHe cP of two lunar samples, [40]

| *T* (K) | c_P_ (J/kg/K) | σ(c_P_) (J/kg/K) |
| --- | --- | --- |
| **sample 10017** | |  |
| 2.344 | 19.33 | 0.46 |
| 2.393 | 19.50 | 0.13 |
| 2.472 | 19.20 | 0.75 |
| 2.713 | 20.21 | 0.17 |
| 2.876 | 20.33 | 0.13 |
| 2.028 | 20.84 | 0.21 |
| 3.399 | 22.13 | 0.21 |
| 3.483 | 22.22 | 0.17 |
| 3.819 | 23.39 | 0.25 |
| 4.036 | 23.93 | 0.21 |
| 4.27 | 25.44 | 0.63 |
| 4.433 | 25.86 | 0.63 |
| 4.525 | 27.15 | 0.29 |
| 4.537 | 27.45 | 0.29 |
| 4.546 | 26.48 | 0.63 |
| 4.708 | 24.94 | 0.63 |
| 4.975 | 28.07 | 0.63 |
|  |  |  |
| **sample 10046** | |  |
| 3.083 | 11.72 | 2.09 |
| 3.268 | 17.57 | 1.67 |
| 3.327 | 14.64 | 2.30 |
| 3.548 | 18.83 | 4.18 |
| 3.717 | 15.90 | 2.09 |
| 4.05 | 10.04 | 1.67 |

## Analogs and lunar reference specific heat

**Table 10 Specific heat capacities of regolith simulants, calculated from c_P_(T) of minerals and composition, assuming mechanical mixture or ideal solid solutions. Jens.Biele@DLR.de, April 25, 2022
Note that the values at high temperatures may not be reproducible, since decomposition of phyllosilicates starts well below 1000K and the notorious epsomite (magnesium sulfate heptahydrate) in the CI simulants starts to loose crystal water already at T>293K. Accuracy is believed to be of order of a few %, except for T<90K where magnetic transition peaks occur; these depend sensitively on whether the minerals are only mechanically mixed (then the curves are correct) or in solid solution (then the endmember peaks tend to blur and shift to lower temperatures). Linear interpolation of the data in this file should reproduce the curves well.**

|  | **cp (J/kg/K)** | | | | | | | |
| --- | --- | --- | --- | --- | --- | --- | --- | --- |
| **T (K)** | **CM-1** | **CM-2** | **CI-1** | **CI-2** | **C2-1** | **CR-1** | **UTPS-TB** | **lunar** |
| 0 | 0 | 0 | 0 | 0 | 0 | 0 | 0 | 0 |
| 2.5 | 0.088199 | 0.0086767 | 0.024995 | 0.019043 | 0.012423 | 0.092583 | 0.006204 | 0.14838 |
| 5 | 0.70191 | 0.065641 | 0.18427 | 0.12613 | 0.084825 | 0.66188 | 0.046509 | 0.83601 |
| 7.5 | 3.8755 | 0.19593 | 0.61896 | 0.40001 | 0.33069 | 1.6614 | 0.17142 | 2.1934 |
| 10 | 8.8118 | 0.41192 | 1.4222 | 0.88048 | 0.74601 | 2.8362 | 0.40628 | 4.2409 |
| 12.5 | 13.312 | 0.73866 | 2.6483 | 1.5861 | 1.2607 | 3.9898 | 0.77091 | 6.9591 |
| 15 | 17.242 | 1.266 | 4.3969 | 2.5916 | 1.9353 | 5.201 | 1.3523 | 10.312 |
| 17.5 | 20.647 | 2.1233 | 6.794 | 3.9942 | 2.8592 | 6.5719 | 2.2939 | 14.256 |
| 20 | 23.971 | 3.4372 | 9.9283 | 5.8889 | 4.133 | 8.2388 | 3.7621 | 18.746 |
| 22.5 | 27.587 | 5.314 | 13.849 | 8.3491 | 5.8337 | 10.341 | 5.8014 | 23.737 |
| 25 | 31.649 | 7.893 | 18.612 | 11.455 | 8.0412 | 12.983 | 8.4913 | 29.185 |
| 27.5 | 36.463 | 11.225 | 24.2 | 15.216 | 10.793 | 16.258 | 11.851 | 35.049 |
| 30 | 42.044 | 15.286 | 30.566 | 19.592 | 14.084 | 20.246 | 15.852 | 41.29 |
| 32.5 | 48.545 | 20.043 | 37.655 | 24.53 | 17.896 | 25.101 | 20.465 | 47.871 |
| 35 | 55.985 | 25.529 | 45.566 | 30.011 | 22.231 | 30.843 | 25.681 | 54.758 |
| 37.5 | 64.453 | 31.775 | 54.321 | 35.989 | 27.062 | 38.297 | 31.45 | 61.92 |
| 40 | 73.447 | 37.848 | 62.349 | 42.416 | 32.359 | 39.656 | 37.721 | 69.329 |
| 42.5 | 83.592 | 43.522 | 66.375 | 49.223 | 38.071 | 45.175 | 44.416 | 76.957 |
| 45 | 94.681 | 50.615 | 74.702 | 56.363 | 44.172 | 51.564 | 51.482 | 84.78 |
| 47.5 | 106.55 | 58.065 | 83.154 | 63.829 | 50.653 | 58.471 | 58.918 | 92.775 |
| 50 | 119.57 | 65.988 | 92.173 | 71.618 | 57.512 | 65.847 | 66.734 | 100.92 |
| 52.5 | 136.7 | 74.305 | 101.52 | 79.727 | 64.849 | 74.034 | 74.926 | 109.2 |
| 55 | 157.46 | 82.913 | 110.95 | 88.114 | 72.631 | 83.128 | 83.431 | 117.59 |
| 57.5 | 176.18 | 91.763 | 120.46 | 96.712 | 80.603 | 92.281 | 92.18 | 126.08 |
| 60 | 186.26 | 100.79 | 130.06 | 105.45 | 88.476 | 100.35 | 101.1 | 134.65 |
| 62.5 | 183.85 | 109.93 | 139.68 | 114.26 | 96.051 | 106.84 | 110.1 | 143.29 |
| 65 | 175.77 | 119.21 | 149.35 | 123.18 | 103.6 | 112.86 | 119.22 | 151.99 |
| 67.5 | 169.57 | 128.72 | 159.16 | 132.31 | 111.44 | 119.25 | 128.58 | 160.73 |
| 70 | 169.61 | 138.53 | 169.16 | 141.71 | 119.77 | 126.77 | 138.23 | 169.5 |
| 80 | 198.58 | 179.92 | 210.51 | 181.21 | 156.16 | 162.14 | 178.91 | 204.75 |
| 90 | 231.96 | 223.44 | 253.06 | 222.7 | 194.92 | 199.64 | 221.62 | 239.88 |
| 100 | 266.6 | 268.22 | 296.35 | 265.49 | 235.22 | 237.98 | 265.45 | 274.51 |
| 110 | 301.42 | 313.48 | 339.95 | 308.95 | 276.38 | 276.68 | 309.58 | 308.39 |
| 120 | 335.83 | 359.04 | 383.85 | 353.01 | 318.34 | 315.17 | 353.77 | 341.35 |
| 130 | 369.18 | 402.9 | 426.16 | 395.37 | 357.7 | 351.34 | 396.32 | 373.28 |
| 140 | 401.43 | 445.24 | 467.08 | 436.23 | 395.25 | 385.87 | 437.33 | 404.09 |
| 150 | 432.52 | 486.29 | 507.01 | 476.05 | 431.82 | 419.25 | 476.98 | 433.75 |
| 160 | 462.3 | 525.91 | 545.8 | 514.66 | 467.16 | 451.2 | 515.12 | 462.25 |
| 170 | 490.74 | 563.97 | 583.34 | 551.93 | 501.06 | 481.65 | 551.64 | 489.59 |
| 180 | 517.85 | 600.42 | 619.56 | 587.78 | 533.47 | 510.5 | 586.56 | 515.79 |
| 190 | 543.65 | 635.28 | 654.48 | 622.23 | 564.44 | 537.88 | 619.88 | 540.87 |
| 200 | 568.28 | 668.57 | 688.07 | 655.25 | 593.93 | 564.19 | 651.64 | 564.87 |
| 210 | 591.71 | 700.33 | 720.39 | 686.88 | 622.04 | 588.85 | 681.9 | 587.81 |
| 220 | 614.01 | 730.6 | 751.48 | 717.16 | 648.76 | 612.3 | 710.71 | 609.75 |
| 230 | 635.3 | 759.47 | 781.39 | 746.15 | 674.2 | 634.85 | 738.16 | 630.71 |
| 240 | 655.61 | 787.03 | 810.27 | 773.99 | 698.54 | 656.26 | 764.52 | 650.75 |
| 250 | 675 | 813.29 | 838.06 | 800.6 | 721.64 | 676.79 | 789.6 | 669.9 |
| 260 | 693.51 | 838.39 | 864.94 | 826.2 | 743.74 | 696.64 | 813.5 | 688.2 |
| 270 | 711.21 | 862.3 | 890.82 | 850.66 | 764.73 | 715.6 | 836.28 | 705.7 |
| 280 | 728.09 | 885.08 | 915.88 | 874.21 | 784.76 | 733.22 | 857.96 | 722.43 |
| 290 | 744.22 | 906.8 | 940.03 | 896.66 | 803.77 | 749.78 | 878.62 | 738.43 |
| 300 | 761.24 | 927.84 | 963.81 | 918.66 | 822.65 | 766.21 | 898.62 | 753.74 |
| 310 | 776.97 | 948 | 986.92 | 939.8 | 840.64 | 781.89 | 917.73 | 768.39 |
| 320 | 792.03 | 967.15 | 1009.4 | 960.15 | 857.61 | 796.63 | 935.91 | 782.42 |
| 330 | 806.47 | 985.54 | 1031.4 | 979.93 | 873.97 | 810.77 | 953.34 | 795.86 |
| 340 | 820.29 | 1003.2 | 1053 | 999.11 | 889.67 | 824.29 | 970.03 | 808.74 |
| 350 | 833.53 | 1020.1 | 1074.2 | 1017.7 | 904.79 | 837.17 | 986.05 | 821.08 |
| 360 | 846.22 | 1036.3 | 1095 | 1035.9 | 919.33 | 849.47 | 1001.4 | 832.92 |
| 370 | 858.37 | 1052 | 1115.4 | 1053.5 | 933.32 | 861.25 | 1016.2 | 844.29 |
| 380 | 870.02 | 1067 | 1135.5 | 1070.6 | 946.79 | 872.59 | 1030.4 | 855.2 |
| 390 | 881.18 | 1081.5 | 1155.3 | 1087.3 | 959.75 | 883.46 | 1044.1 | 865.68 |
| 400 | 891.73 | 1094.9 | 1174.5 | 1103.2 | 971.99 | 893.84 | 1056.8 | 875.76 |
| 410 | 901.72 | 1107.4 | 1193.1 | 1118.4 | 983.59 | 903.73 | 1068.7 | 885.45 |
| 420 | 911.28 | 1119.4 | 1211.5 | 1133.2 | 994.73 | 913.23 | 1080.1 | 894.78 |
| 430 | 920.43 | 1130.9 | 1229.6 | 1147.5 | 1005.4 | 922.34 | 1091 | 903.76 |
| 440 | 929.2 | 1142 | 1247.5 | 1161.5 | 1015.7 | 931.14 | 1101.5 | 912.42 |
| 450 | 937.61 | 1152.7 | 1265.2 | 1175.1 | 1025.5 | 939.62 | 1111.7 | 920.77 |
| 460 | 945.67 | 1163 | 1282.7 | 1188.4 | 1035 | 947.78 | 1121.4 | 928.82 |
| 470 | 953.41 | 1172.9 | 1300 | 1201.4 | 1044.1 | 955.63 | 1130.8 | 936.59 |
| 480 | 960.83 | 1182.5 | 1317.2 | 1214.1 | 1052.8 | 963.19 | 1139.9 | 944.1 |
| 490 | 967.97 | 1191.8 | 1334.3 | 1226.5 | 1061.3 | 970.48 | 1148.7 | 951.36 |
| 500 | 974.84 | 1200.9 | 1351.3 | 1238.8 | 1069.5 | 977.49 | 1157.3 | 958.39 |
| 520 | 987.84 | 1218.1 | 1385.2 | 1263 | 1085.1 | 990.72 | 1173.6 | 971.78 |
| 540 | 999.96 | 1234.5 | 1419.2 | 1286.6 | 1100 | 1003.2 | 1189.1 | 984.36 |
| 560 | 1011.3 | 1250.2 |  |  | 1114.2 | 1015.4 | 1203.9 | 996.22 |
| 580 | 1022 | 1265.2 |  |  | 1127.9 | 1026.8 | 1218.1 | 1007.4 |
| 600 | 1032.2 | 1279.8 |  |  | 1141.2 | 1037.7 | 1231.8 | 1018.1 |
| 620 | 1041.8 | 1293.9 |  |  | 1154.3 | 1048.3 | 1245 | 1028.2 |
| 640 | 1050.9 | 1307.7 |  |  | 1167.1 | 1058.5 | 1257.9 | 1037.8 |
| 660 | 1059.5 | 1321.1 |  |  | 1179.9 | 1068.6 | 1270.5 | 1047 |
| 680 | 1067.6 | 1334.3 |  |  | 1192.6 | 1078.9 | 1282.7 | 1055.9 |
| 700 | 1075.4 | 1347.3 |  |  | 1205.4 | 1089.2 | 1294.8 | 1064.4 |
| 720 | 1082.9 | 1360.3 |  |  | 1218.5 | 1100.4 | 1306.8 | 1072.6 |
| 740 | 1090 | 1373.2 |  |  | 1231.9 | 1110.9 | 1318.7 | 1080.6 |
| 760 | 1096.9 | 1386.2 |  |  | 1245.9 | 1122.3 | 1330.6 | 1088.3 |
| 780 | 1103.6 | 1399.4 |  |  | 1260.4 | 1133.7 | 1342.6 | 1095.8 |
| 800 | 1110.1 | 1412.8 |  |  | 1275.8 | 1145.8 | 1354.7 | 1103.1 |
| 820 | 1116.7 |  |  |  | 1295.8 | 1160.2 | 1368.4 | 1110.3 |
| 840 | 1123.5 |  |  |  | 1326 | 1181.1 | 1385.7 | 1117.3 |
| 860 | 1126.7 |  |  |  | 1278.9 | 1153.7 | 1375 | 1124.2 |
| 880 | 1131.3 |  |  |  | 1268.2 | 1149 | 1377.4 | 1131 |
| 900 | 1136.2 |  |  |  | 1265.9 | 1149.9 | 1382.7 | 1137.7 |
| 920 | 1141.1 |  |  |  | 1266.2 | 1158.4 | 1388.8 | 1144.3 |
| 940 | 1146 |  |  |  | 1268.5 | 1172.3 | 1395.6 | 1150.9 |
| 960 | 1150.8 |  |  |  | 1271.9 | 1177.9 | 1402.7 | 1157.4 |
| 980 | 1155.6 |  |  |  | 1275.8 | 1195.6 | 1409.9 | 1163.9 |
| 1000 | 1160.2 |  |  |  | 1280.2 | 1176.7 | 1417.3 | 1170.3 |

# Mineral composition of analogue materials

Commercial asteroid simulants from the now defunct private company Deep Space Industries, USA [https://deepspaceindustries.com/simulants/ - Deep Space Industries was acquired on January 1, 2019 by Bradford Space[[3]](#footnote-3) .

An iron/nickel mixture consisting of 93% Fe, 7% Ni is used. Sub-bituminous coal is a kerogen substitute.

Note that in order to find the correct mass fractions of endmember minerals in solid solutions (like olivine, or (ortho-)pyroxenes listed here, one need to convert the given atomic fractions into mass fractions (equations ,).

## CI simulants

Orgueil-type CI. Available are UCF/DSI-CI-**1** (better elemental fidelity, especially volatiles) and UCF/DSI-CI-**2** (better mineral fidelity, appearance and stability) with the following spec sheets (**Table 11**-Table 16)

**Table 11 UCF/DSI-CI-1 Orgueil simulant mineralogical composition**

| **Mineral** | Weight % | Notes |
| --- | --- | --- |
| **Antigorite** | 48.0% | A serpentine mineral, (Mg,Fe^++^)3Si_2_O_5_(OH)_4_ |
| **Epsomite** | 6.0% | Magnesium sulfate heptahydrate MgSO_4_⋅7H_2_O |
| **Magnetite** | 13.5% | Iron Oxide – Fe_3_O_4_ (actually present 14.5%) |
| **Attapulgite** | 5.0% | AKA palygorskite, (Mg,Al)_2_Si_4_O_10_(OH)·4(H_2_O) This clay binds strongly, without swelling/shrinking |
| **Olivine** | 7.0% | Magnesium Iron Silicate – (Mg_0.9_ Fe_0.1_)_2_SiO_4_ |
| **Pyrite** | 6.5% | Iron Sulfide (FeS_2_) substituted for troilite (FeS) |
| **Vermiculite** | 9.0% | A smectite-group clay (Mg,Fe,Al)_3_(Al,Si)_4_O_10_(OH)_2_ - 4H_2_O |
| **Coal** | 5.0% | Sub-bituminous coal is a kerogen substitute |

**Table 12 UCF/DSI-CI-2 Orgueil simulant mineralogical composition**

| **Mineral** | Weight % | Notes |
| --- | --- | --- |
| **Antigorite** | 36.5% | A serpentine mineral, (Mg,Fe^++^)3Si2O5(OH)4 |
| **Epsomite** | 15.0% | Magnesium sulfate heptahydrate – MgSO4⋅7H2O |
| **Magnetite** | 11.5% | Iron Oxide – Fe3O4 (actually present 14.5%) |
| **Attapulgite** | 9.0% | AKA palygorskite, (Mg,Al)2Si4O10(OH)·4(H2O) This clay binds strongly without swelling/shrinking |
| **Olivine** | 7.0% | Magnesium Iron Silicate – (Mg0.9Fe0.1)2SiO4 |
| **Pyrite** | 6.0% | Iron Sulfide (FeS2) substituted for troilite (FeS) |
| **Vermiculite** | 5.0% | A smectite-group clay (Mg,Fe,Al)3(Al,Si)4O10(OH)2 - 4H2O |
| **Siderite** | 4.0% | Iron Carbonate – FeCO3 |
| **Coal** | 3.5% | Sub-bituminous coal is a kerogen substitute |
| **Gypsum** | 2.5% | Calcium Sulfate Di-hydrate – CaSO4⋅2H2O |

## CM simulants

Like the CI-type carbonaceous chondrites, the CMs are dominated by phyllosilicates. The major difference is the predominance of iron rich serpentine polymorph cronstedtite.  Available are UCF/DSI-CM-1 (best appearance and physical characteristics) and UCF/DSI-CM-2 (improved mineralogy and volatiles content).

**Table 13 UCF/DSI-CM-1 Murchinson simulant mineralogical Composition**

| **Mineral** | Weight % | Notes |
| --- | --- | --- |
| **Ferrous Silicate** | 57.0% | An iron rich silicate (Fe2SiO4) aka Fayalite or “copper slag grit” substituted for the serpentine mineral Cronstedtite, (Fe^++^2,Fe^+++^)(Si,Fe^+++^)O5(OH)4 |
| **Antigorite** | 22.0% | A serpentine mineral, (Mg,Fe^++^)3Si2O5(OH)4 |
| **Olivine** | 8.1% | Magnesium Iron Silicate – (Mg0.9Fe0.1)2SiO4 |
| **Coal** | 3.5% | Sub-bituminous coal is a kerogen substitute |
| **Pyrite** | 2.5% | Iron Sulfide (FeS2), substituted for troilite (FeS) |
| **Pyroxene** | 2.0% | Mg0.75Fe0.25SiO3 |
| **Magnetite** | 1.0% | Iron Oxide Fe3O4 |
| **Dolomite** | 1.0% | (CaMg)[CO3]2 |
| **Sodium Silicate** | 2.9% | Note that 5.0% of sodium silicate pentahydrate is added, but  the water is driven out by the lithification process |

**Table 14 UCF/DSI-CM-2 Murchison simulant mineralogical composition**

| **Mineral** | Weight % | Notes |
| --- | --- | --- |
| **Antigorite** | 70.0% | A serpentine mineral, (Mg,Fe++)3Si2O5(OH)4 |
| **Magnetite** | 10.0% | Iron Oxide – Fe3O4 |
| **Olivine** | 7.5% | Magnesium Iron Silicate – (Mg0.9Fe0.1)2SiO4 |
| **Coal** | 3.5% | Sub-bituminous coal is a kerogen substitute |
| **Pyrite** | 2.5% | Iron Sulfide (FeS2), substituted for troilite (FeS) |
| **Pyroxene** | 2.0% | Mg0.75Fe0.25SiO3 |
| **Sodium Silicate** | 3.5% | Note that 6.0% of sodium silicate pentahydrate is added, but the water is driven out by the lithification process |
| **Siderite** | 1.0% | FeCO3 |

## C2 type simulant

Tagish Lake-type C2 Carbonaceous Chondrite simulant. This material sampled a different mixture of source materials than the CIs.  It is more olivine and magnetite rich while being a little depleted in serpentine relative to CIs. Available as UCF/DSI-C2-1.

**Table 15 UCF/DSI-C2-1 Tagish Lake Simulant mineralogical composition**

| **Mineral** | Weight % | Notes |
| --- | --- | --- |
| **Lizardite** | 30.5% | A serpentine mineral, Mg3Si2O5(OH)4 |
| **Olivine** | 25.0% | Magnesium Iron Silicate – (Mg0.9Fe0.1)2SiO4 |
| **Magnetite** | 22.0% | Iron Oxide – Fe3O4 |
| **Pyrite** | 8.5% | Iron Sulfide (FeS2) |
| **Coal** | 5.0% | Sub-bituminous coal is a kerogen substitute |
| **Vermiculite** | 4.0% | A smectite-group clay (Mg,Fe,Al)3(Al,Si)4O10(OH)2 ⋅ 4H2O |
| **Attapulgite** | 4.0% | aka palygorskite, (Mg,Al)2Si4O10(OH)·4(H2O) This clay binds strongly without swelling/shrinking |
| **Dolomite** | 1.0% | Calcium Magnesium Carbonate – CaMg(CO3)2 |

## CR simulant

CRs are less “primitive” than CMs and CIs, have lower clay contents and thus lower volatile contents, more FeNi free metal, and have more mafic silicates. As such they seem to represent an intermediate group in the spectrum of volatile rich to volatile poor carbonaceous chondrites. The most pristine CR falls are also the most anomalous members of this group (and may not really belong in the group). DSI uses a collection of five low-to-moderate weathering Antarctic finds that have mineralogies roughly average for the overall group as guide for the CR recipe.

Table 16 UCF/DSI-CR-1 Simulant Mineralogical composition

| **Mineral** | Weight % | Notes |
| --- | --- | --- |
| **Antigorite** | 9.0% | A serpentine mineral, (Mg,Fe^2+^)3Si2O5(OH)4 |
| **Pyroxene** | 31.0% | Mg0.75Fe0.25SiO3 |
| **Magnetite** | 14.0% | Iron Oxide – Fe3O4 |
| **Iron-Nickel** | 5.0% | An iron/nickel mixture consisting of 93% Fe, 7% Ni |
| **Olivine** | 33.0% | Magnesium Iron Silicate – (Mg0.9Fe0.1)2SiO4 |
| **Pyrite** | 4.0% | Iron Sulfide (FeS2), substituted for troilite (FeS) |
| **Sodium Silicate** | 2.0% | Note that 3.5% of sodium silicate pentahydrate is added, but the water is driven out by the lithification process |
| **Coal** | 2.0% | Sub-bituminous coal is a kerogen substitute |

## UTPS-TB Phobos simulant

This is one of the the University of Tokyo Phobos simulant, Tagish Lake Variant by Hideaki Miyamoto and Takafumi Niihara, [41].

The mineral composition is approximately (Miyamoto, priv. comm.),

Serpentine 62.5%

Magnetite 7.9%

Pyrite 9.4%

Olivine 7.6%

Limestone 4.6%

Dolomite 4.7%

Organic materials 3.3%

We took antigorite as the serpentine; an olivine with Fo_90_Fa_10_ and sub-bitumous coal as "organic material".

In Figure 8 we plot the estimated UTPS-TB specific heat together with the measured *c_P_* of a Martian shergottite (basaltic).

Figure 8 Comparison of specific heat capacity of possible Phobos surface materials. Blue curve, calculated *c_P_* of Phobos surface simulant UTPS-TB, based on Tagish Lake meteorite (which most likely originated from 773 Irmintraud, a D-type asteroid). Black curve, measured $\boldsymbol{c}_{\boldsymbol{p}}$ of Martian meteorite Los Angeles after [27].

# Supporting data and figures for the bronzite mixing example

The bronzite sample was described by [42] as follows: “We measured three perfect, gem-quality, centimeter-sized orthopyroxene single crystals that are similar except for their minor element chemistries, principally the chromium and aluminum concentrations (Table1). Multiple spot analyses of each chip revealed no chemical inhomogeneities within any single chip. The crystals ranged mass from 0.19 to 1.65 g. The crystals were carefully characterized by optical, microscopy, X ray diffraction, and electron microprobe techniques. Unit cell dimensions are included in Table 1. Each crystal is of a single phase, free of exsolution lamellae, and chemically homogeneous (Figure 1). .. Electron microscopy reveals that the crystals are remarkably devoid of defects and contain no Gunier-Preston zones”.

From the chemical analysis given in [43], together with the compositions of the three chips given in table 1 of [42] we estimated (mole fractions normalized to Mg+Fe+Ca=1 **and** Si=1; see Table 17 and Table 18) the following empirical formula of the orthopyroxene, the remaining impurities and the corresponding 1-σ uncertainties of the stoichiometric coefficients:

Mg_x1_Fe_x2_Ca_x3_SiO_3_ + Al_0.014_Mn_0.003_Cr_0.001_O_0.026_

x1=0.843±0.028, x2=0.151±0.021, x3=0.006±0.007. Molar mass 105.23 g/mol.

The impurities correspond to about 0.008 Al_2_O_3_ + 0.003 MnO + 0.0008 Cr_2_O_3_ and are neglected.

**Figure 9 Bronzite [43] relative deviations observed Cp minus calculated Cp; improved fit allowing Mg and Fe (En and Fs) mole fractions to be free. Data <100 K were excluded from the fit. Relative deviations are less than 1% outside of the Schottky peak regions (10-100 K). The fit used weighting with 1% relative uncertainty for all data points; the fit equation is Cp(T) = (x1*cp_mineral(T,'En')*0.100389+x2*cp_mineral(T,'Fs')*0.131931) where the numerical coefficients are just the molar masses of En and Fs. At 12 K, the relative deviation is 46%, at 38 K, 67%.**

Table 17 Bronzite compositions, chemical analysis [42, 43]

| 3 chips; OxideWeightPercent | CationsPerSixOxygens | | | |
| --- | --- | --- | --- |
| Table 1 of HUEBNER ET AL. 1979 | | |  |
|  |  |  |  |
|  | MF-1 | MF-2 | MF-3 |
| **SiO2** | 55.34 | 55.71 | 57.07 |
| **Al_2_O_3_** | 1.89 | 1.3 | 0.55 |
| **Cr_2_O_3_** | 0.51 | 0.07 | 0.02 |
| **FeO** | 10.56 | 11.57 | 8.51 |
| **MgO** | 30.61 | 30.45 | 33.18 |
| **MnO** | 0.18 | 0.2 | 0.16 |
| **CaO** | 0.23 | 0.21 | 0.19 |
| **TiO2** | 0.02 | 0.04 | 0 |
| **Na2O** | 0.01 | 0 | 0 |
|  |  |  |  |
| Composition Krupka 1985a, oxide weight percents | | | |
| **SiO2** | 55.86 |  |  |
| **Al_2_O_3_** | 0.66 |  |  |
| **Cr_2_O_3_** | 0.1 |  |  |
| **FeO** | 10.11 |  |  |
| **MgO** | 31.61 |  |  |
| **MnO** | 0.19 |  |  |
| **CaO** | 0.29 |  |  |
| **Total** | 98.82 |  |  |

Table 18 Bronzite empirical formula results, various normalisations

| **Empirical formulae, normalized to ..** | | |  |  | **3O** | | **1Si** | | **Mg+Fe+Ca=1** | | **(Mg+Fe+Ca=1) -Mg*En-Fe*Fs** | | **Normalize rest  to 1O** | |
| --- | --- | --- | --- | --- | --- | --- | --- | --- | --- | --- | --- | --- | --- | --- |
|  | **Krupka** | **Huebner MF1** | **Huebner MF2** | **Huebner MF3** | **Mean** | **Std** | **Mean** | **Std** | **Mean** | **Std** | **Mean** | **Std** |  |  |
| **Element** | **mole fraction normalized to 3O** | | | |  |  |  |  |  |  |  |  |  |  |
| Si | 0.9913 | 0.9795 | 0.9874 | 0.9951 | **0.9883** | **0.0066** | **1.0000** | **0.0067** | **1.0059** | **0.0068** | 0.0059 | 0.0068 | 0.1100 |  |
| O | 3.0000 | 3.0000 | 3.0000 | 3.0000 | **3.0000** | 0.0000 | **3.0355** | 0.0000 | **3.0532** | 0.0000 | **0.0532** | 0.0000 | 1.0000 |  |
| Al | 0.0138 | 0.0394 | 0.0272 | 0.0113 | **0.0229** | **0.0130** | **0.0232** | **0.0132** | 0.0233 | 0.0133 | **0.0233** | **0.0133** | 0.4384 |  |
| Cr | 0.0014 | 0.0071 | 0.0010 | 0.0003 | 0.0024 | 0.0032 | 0.0025 | 0.0032 | 0.0025 | 0.0032 | 0.0025 | 0.0032 | 0.0468 |  |
| Fe | 0.1500 | 0.1563 | 0.1715 | 0.1241 | **0.1505** | **0.0198** | **0.1523** | **0.0200** | **0.1532** | **0.0201** | 0.0000 | 0.0201 | 0.0000 |  |
| Mg | 0.8362 | 0.8077 | 0.8045 | 0.8625 | **0.8277** | **0.0272** | **0.8375** | **0.0275** | **0.8424** | **0.0277** | 0.0000 | 0.0277 | 0.0000 |  |
| Mn | 0.0029 | 0.0027 | 0.0030 | 0.0024 | 0.0027 | 0.0003 | 0.0028 | 0.0003 | 0.0028 | 0.0003 | **0.0028** | **0.0003** | 0.0522 |  |
| Ca | 0.0055 | 0.0044 | 0.0040 | 0.0035 | **0.0044** | **0.0008** | **0.0044** | **0.0009** | **0.0044** | **0.0009** | 0.0000 | 0.0009 | 0.0000 |  |
| Ti | 0.0000 | 0.0003 | 0.0005 | 0.0000 | 0.0002 | 0.0003 | 0.0002 | 0.0003 | 0.0002 | 0.0003 | 0.0002 | 0.0003 | 0.0038 |  |
| Na | 0.0000 | 0.0002 | 0.0000 | 0.0000 | 0.0000 | 0.0001 | 0.0000 | 0.0001 | 0.0000 | 0.0001 | 0.0000 | 0.0001 | 0.0008 |  |
|  |  |  |  |  |  |  |  |  |  |  |  |  |  |  |
| sum | 5.0011 | 4.9976 | 4.9991 | 4.9991 | **4.9992** | **0.0014** |  |  |  |  |  |  |  |  |
| M | 105.3657 | 105.6447 | 105.9951 | 104.4292 | **105.3587** | **0.6710** |  |  |  |  |  |  |  |  |

# References

1. Goetsch, R., et al., *Structural, thermal, magnetic, and electronic transport properties of the LaNi 2 (Ge 1− x P x) 2 system.* Physical Review B, 2012. **85**(5): p. 054517.

2. Gurevich, V., et al., *Heat capacity and thermodynamic functions of epsomite MgSO 4· 7 H 2 O at 0–303 K.* Geochemistry International, 2007. **45**(2): p. 206-209.

3. Efron, B. and G. Gong, *A Leisurely Look at the Bootstrap, the Jackknife, and Cross-Validation.* The American Statistician, 1983. **37**(1): p. 36-48.

4. Opeil, C.P., et al., *Stony meteorite thermal properties and their relationship to meteorite chemical and physical states.* Meteorit Planet Sci, 2012. **47**, DOI: 10.1111/j.1945-5100.2012.01331.x.

5. Consolmagno, G.J., et al., *The measurement of meteorite heat capacity at low temperatures using liquid nitrogen vaporization.* Planetary and Space Science, 2013. **87**: p. 146-156.

6. Consolmagno, G., R. Macke, and D. Britt. *Meteorite heat capacities: Results to date*. in *Asteroids, Comets, Meteors 2014*. 2014.

7. Macke, R., G. Consolmagno, and D. Britt. *Heat Capacity Measurements of HED Meteorites from the Vatican Collection*. in *Lunar and Planetary Science Conference*. 2014.

8. Macke, R., et al. *Ordinary Chondrite Heat Capacities Below 350K*. in *Lunar and Planetary Science Conference*. 2016.

9. Macke, R., C. Opeil, and G. Consolmagno. *Quantifying Weathering in Ordinary Chondrite Finds Using Heat Capacity*. in *Lunar and Planetary Science Conference*. 2017.

10. Bonidie, M., et al., *Physical and Thermal Properties of Iron Meteorites below 300 K.* Bulletin of the American Physical Society, 2019.

11. Macke, R.J., et al., *Heat capacities of ordinary chondrite falls below 300 K.* 2019. **54**(11): p. 2729-2743.

12. Opeil, C., et al., *The surprising thermal properties of CM carbonaceous chondrites.* Meteoritics & Planetary Science, 2020. **55**(8).

13. Piqueux, S., et al., *Specific Heat Capacity Measurements of Selected Meteorites for Planetary Surface Temperature Modeling.* Journal of Geophysical Research: Planets, 2021. **126**(11): p. e2021JE007003.

14. Rubin, A.E., et al., *Los Angeles: The most differentiated basaltic martian meteorite.* Geology, 2000. **28**(11): p. 1011-1014.

15. Warren, P.H., J.P. Greenwood, and A.E. Rubin, *Los Angeles: A tale of two stones.* Meteoritics & Planetary Science, 2004. **39**(1): p. 137-156.

16. Butler, C.P. and R.J. Jenkins, *Thermal Properties of Meteoritic Iron from-150° to 300° Celsius.* Science, 1963. **139**(3554): p. 486-487.

17. Matsui, T. and M. Osako, *Thermal property measurement of Yamato meteorites.* 1979.

18. Yomogida, K. and T. Matsui, *Physical properties of ordinary chondrites.* Journal of Geophysical Research: Solid Earth, 1983. **88**(B11): p. 9513-9533.

19. Soini, A.J., et al., *Thermal and porosity properties of meteorites: A compilation of published data and new measurements.* 2020.

20. Beech, M., et al., *The thermal and physical characteristics of the Gao-Guenie (H5) meteorite.* Planetary and Space Science, 2009. **57**(7): p. 764-770.

21. Flynn, G.J., et al., *Physical properties of the stone meteorites: Implications for the properties of their parent bodies.* Chemie der Erde, 2018. **78**: p. 269–298, DOI: https://doi.org/10.1016/j.chemer.2017.04.002.

22. Szurgot, M. *On the specific heat capacity and thermal capacity of meteorites*. in *Lunar and Planetary Science Conference*. 2011.

23. Opeil, C.P., G.J. Consolmagno, and D.T. Britt, *The thermal conductivity of meteorites: New measurements and analysis.* Icarus, 2010. **208**(1): p. 449-454, DOI: https://doi.org/10.1016/j.icarus.2010.01.021.

24. Łuszczek, K. and R.A. Wach, *NWA 6255 meteorite− Thermophysical properties of interior and the crust.* Meteorites, 2014. **3**.

25. Szurgot, M., et al., *Investigation of microstructure and thermophysical properties of Morasko iron meteorites.* Crystal Research and Technology: Journal of Experimental and Industrial Crystallography, 2008. **43**(9): p. 921-930.

26. Yu, L. and J. Ji, *Surface thermophysical properties determination of OSIRIS-REx target asteroid (101955) Bennu.* Monthly Notices of the Royal Astronomical Society, 2015. **452**(1): p. 368-375.

27. OPEIL SJ, C., et al., *Stony meteorite thermal properties and their relationship with meteorite chemical and physical states.* Meteoritics & Planetary Science, 2012. **47**(3): p. 319-329.

28. Gundlach, B. and J. Blum, *A new method to determine the grain size of planetary regolith.* Icarus, 2013. **223**(1): p. 479-492.

29. Ghosh, A. and H.Y. McSween, *Temperature dependence of specific heat capacity and its effect on asteroid thermal models.* Meteoritics & Planetary Science, 1999. **34**(1): p. 121-127, DOI: 10.1111/j.1945-5100.1999.tb01737.x.

30. Henke, S., et al., *Thermal evolution and sintering of chondritic planetesimals.* Astronomy & Astrophysics, 2012. **537**: p. A45.

31. Jarosewich, E., *Chemical analyses of meteorites: A compilation of stony and iron meteorite analyses.* Meteoritics & Planetary Science, 1990. **25**(4): p. 323-337.

32. Barin, I., *Thermochemical data of pure substances*. 3rd ed. Vol. 304. 1995: Wiley Online Library.

33. SZURGOT, M., R.A. WACH, and T.A. PRZYLIBSKI, *Thermophysical properties of the Sołtmany meteorite.* Meteorites, 2012. **2**(1-2): p. 53-65.

34. Hemingway, B.S., R.A. Robie, and W.H. Wilson, *Specific heats of lunar soils, basalt, and breccias from the Apollo 14, 15, and 16 landing sites, between 90 and 350 K*, in *Proceedings of the fourth lunar science conference (Supplement 4, Geochimia et Cosmochimica Acta)* 1973. p. 2481-2487.

35. Robie, R., B. Hemingway, and W. Wilson. *Specific heats of lunar surface materials from 90 to 350 K*. in *Proceedings of the Apollo 11 Lunar Science Conference held 5-8 January, 1970*. 1970. Houston, TX: Geochimica et Cosmochimica Acta Supplement 1.

36. Robie, R. and B. Hemingway. *Specific heats of the lunar breccia (10021) and olivine dolerite (12018) between 90° and 350° Kelvin*. in *Lunar and Planetary Science Conference Proceedings*. 1971. Houston, TX: The M.I.T. press.

37. Horai, K., et al., *Thermal diffusivity, conductivity and thermal inertia of Apollo 11 lunar material.* Proceedings fo the Apollo 11 Lunar Science Conference, (Geochimica et Cosmochimica Acta Supplement), 1970. **3**: p. 2243-2249.

38. Heiken, G., D. Vaniman, and B.M. French, *Lunar sourcebook - A user's guide to the Moon*. 1991, Cambridge: Cambridge University Press. 736.

39. Cremers, C.J., *Heat Transfer Within the Lunar Surface Layer*, in *Advances in Heat Transfer*, J.P. Hartnett and T.F. Irvine, Editors. 1974, Elsevier. p. 39-83, DOI: https://doi.org/10.1016/S0065-2717(08)70109-6.

40. Morrison, J. and P. Norton, *The heat capacity and thermal conductivity of Apollo 11 lunar rocks 10017 and 10046 at liquid helium temperatures.* Journal of Geophysical Research, 1970. **75**(32): p. 6553-6557.

41. Miyamoto, H., et al., *Phobos Environment Model and Regolith Simulant for MMX Mission.* 2018.

42. Huebner, J., A. Duba, and L. Wiggins, *Electrical conductivity of pyroxene which contains trivalent cations: Laboratory measurements and the lunar temperature profile.* Journal of Geophysical Research: Solid Earth, 1979. **84**(B9): p. 4652-4656, DOI: https://doi.org/10.1029/JB084iB09p04652.

43. Krupka, K.M., et al., *Low-temperature heat capacities and derived thermodynamic properties of anthophyllite, diopside, enstatite, bronzite, and wollastonite.* American Mineralogist, 1985a. **70**(3-4): p. 249-260.

1. as used in calorimetry [↑](#footnote-ref-1)
2. Applying the curvature correction to the mean Consolmagno & Macke LN2 values, the real *c_P_* at the average temperature of 185.5K is about 4.1% higher (see section 1.4); the average *c_P_* rather corresponds to a notional temperature of 177.3 K (this latter value is only exact for the lunar *c_P_*(*T*) curve; Consolmagno and Macke cite 175K at the notional temperature, quite close). The *c_P_* data measured with the LN2 drop calorimetry have random and systematic uncertainties of ~2% and ~2%, respectively. [↑](#footnote-ref-2)
3. https://www.bradford-space.com; https://spacenews.com/deep-space-industries-acquired-by-bradford-space/ [↑](#footnote-ref-3)
